# Effects of attractions and social attributes on peoples’ usage intention and media dependence towards chatbot: The mediating role of parasocial interaction and emotional support

**DOI:** 10.1186/s40359-025-03284-w

**Published:** 2025-08-29

**Authors:** Ke Zhang, Yuchen Xie, Du Chen, Zhouyu Ji, Jing Wang

**Affiliations:** 1 School of Communication, Soochow University, Suzhou, Jiangsu China; 2 College of Arts & Media, Tongji University, Shanghai, China; 3 City Culture and Communication College, Suzhou City University, Suzhou, Jiangsu China

**Keywords:** Attraction, Social attribute, Para-social interaction, Emotional support, Usage intention, Media dependency

## Abstract

**Purpose:**

It is important to explore the relationship between humans and chatbots to improve human–robot interaction in the era of artificial intelligence. This study aims to explore the effects of attractions and social attributes of chatbots on users’ media dependency and usage intention of chatbots, as well as the role of users’ para-social interaction and emotional support gained from chatbots.

**Methods:**

A total of 1,553 responses were collected based on a cross-sectional online survey. Utilizing the structural equation modeling approach, this study tested the relationships among exogenous variables (social attraction/task attraction, perceived competence/perceived warmth), endogenous variables (usage intention/media dependence), and mediating variables (para-social interaction/emotional support).

**Results:**

The results show that the attraction and social attributes of chatbots, represented by ChatGPT, enable users to construct para-social interaction and obtain emotional support when chatting with them. Meanwhile, para-social interaction and emotional support can link users’ perceptions of chatbots to their media dependency on and usage intention of them. This study provides theoretical and methodological references for examining the human–robot interaction relationship and offers insights into exploring the human–robot emotional connection.

**Conclusions:**

This study explores chatbots from the perspective of emotional connections, emphasizing how users’ perceptions of chatbot attraction and social attributes facilitate para-social interaction and emotional support. Theoretically, it extends the application of para-social interaction theory and emotional support into the domain of human-chatbot communication, enriching the understanding of affective mechanisms in human-AI relationships. Methodologically, the study employs structural equation modeling (SEM) to test a multidimensional mediation model using large-scale survey data, examining psychological pathways linking chatbot characteristics to users' behavioral responses. These findings offer new insights for the optimization of human–computer interaction applications and the improvement of chatbot design in practice.

## Introduction

Chatbot is a computer program, which responds like a smart entity when conversed with through text or voice and understands one or more human languages by Natural Language Processing (NLP) Khanna et al. [30] Chatbots are also known as smart bots, interactive agents, digital assistants, or artificial conversation entities, which evolved rapidly in numerous fields in recent years, including marketing, supporting systems, education, health care, cultural heritage, and entertainment [2].

Many scholars have researched the relevant technology of chatbots and tried to find a better design for chatbots [8, 50]. With the gradual development and maturity of this technology, scholars have also noticed that it has had a profound application in many aspects of human daily life. For instance, the research results of Liu et al. provide insights into how a chatbot with AI techniques can create a positive reading experience to sustain students’ interest in learning Liu et al. [35] Thus, scholars have begun to pay attention to the complex interaction between humans and robots. In the field of human–robot interaction, emotionalization has always been the focus of research [9]. In the process of human–robot interaction, emotional elements such as empathy, emotional feedback, and affect recognition are indispensable for promoting effective communication and relational trust [46, 67] and may influence users’ trust on robots [17].

Since the end of 2022, ChatGPT has hit the big time across the globe. ChatGPT not only has a profound impact on many industries but also triggers a heated debate in academia. As a relatively new technology, most research focuses on the functions of ChatGPT and its impacts. For example, Taecharungroj analyzed 233,914 English tweets about “What can ChatGPT do?” and identified five functional domains: creative writing, essay writing, prompt writing, code writing, and answering questions [64]. Compared with earlier generations of task-based bots, ChatGPT demonstrates more advanced emotional engagement capabilities—it can simulate empathy, maintain contextual memory, and produce emotionally resonant responses through human-like dialogue. Recent studies have begun to document users'affective experiences when interacting with ChatGPT, highlighting its capacity to evoke satisfaction, enhance writing confidence, and support emotionally resonant communication in academic contexts [31].These emotional capacities have prompted both public fascination and academic reflection, especially after incidents such as the viral New York Times interview in which Bing (powered by ChatGPT) expressed love for a journalist. Such interactions highlight the increasing blurring of boundaries between social machines and human partners.

Recent breakthroughs in the anthropomorphic emotional expression of chatbots—particularly those powered by GPT-based systems—have significantly advanced their ability to simulate empathy, convey warmth, and foster emotionally engaging interactions. While these developments have sparked widespread public and academic interest, empirical studies on the mechanisms through which users form emotional connections with chatbots remain limited. Although some recent research has explored empathy in AI-human interaction—for example, showing how AI companions like Replika support affective coping and psychological resilience during the COVID-19 pandemic [26]—systematic examination of the psychological mechanisms behind emotional bonding with chatbots like ChatGPT is still lacking. Prior research has predominantly emphasized technical performance or cognitive trust, often overlooking the affective and relational pathways that underlie users’ ongoing engagement and dependency in everyday contexts.

To address this gap, the present study draws on four complementary theoretical frameworks to construct an integrated model of human–chatbot emotional connection. Social Attractiveness Theory explains how users’ perceptions of a chatbot’s task attraction (usefulness and task performance) and social attraction (friendliness and likeability) motivate interaction. The Stereotype Content Model (SCM) provides a framework for understanding social attributes, defined here as perceived competence (e.g., intelligence, reliability) and perceived warmth (e.g., sincerity, empathy), which shape users’ social evaluations of chatbots. Para-social Interaction Theory helps explain how users form one-sided but emotionally meaningful relationships with chatbots, while Social Support Theory—specifically its dimension of emotional support—accounts for the perceived empathy, care, and emotional reassurance users receive during interactions.

By combining these perspectives, the study explores the psychological mechanisms that link chatbot characteristics (attraction and social attributes) to user outcomes, specifically media dependency (users’ reliance on chatbots for emotional and informational needs) and usage intention (users’ willingness to continue engaging with chatbots). These relationships are further examined through the mediating roles of para-social interaction and emotional support. Based on this framework, the study addresses the following research questions:

- Q1: How do users’ perceptions of chatbot attractions (i.e., social and task-based) and social attributes (i.e., perceived warmth and competence) influence their media dependency and usage intention?
- Q2: How do para-social interaction and perceived emotional support mediate the relationship between chatbot characteristics and users’ media dependency and usage intention?

 

## Literature Review

The core of current research on interaction design lies in users’ experience [69]. Therefore, this study first examines the interaction design of chatbots and introduces attraction and robotic social attributes. Based on that, this study constructs a model that the emotional connection between chatbots and users affects users’ media dependency and intention to use.

### Attractions of Chatbots, Parasocial Interaction and Emotional Support of Chatbots

McCroskey and McCain pointed out that interpersonal attraction consists of three dimensions: task attraction, social attraction, and physical attraction [38]. Task attraction refers to an individual’s desire to work with another based on perceived competence and effectiveness in accomplishing tasks, while social attraction involves the extent to which an individual is considered likable and pleasant to interact with. Physical attraction, on the other hand, relates to the degree of positive evaluation one holds toward another’s physical appearance, including facial features and body proportions. Physical attraction is excluded from the current framework because chatbots like ChatGPT are text-based and do not possess visually embodied forms. Given the lack of visual presence or anthropomorphic appearance cues, it is methodologically inappropriate and conceptually irrelevant to measure users’ physical attraction toward such non-visual agents. Therefore, the study centers on the two applicable dimensions—task and social attraction—to reflect the interpersonal aspects of user-chatbot interaction in a text-based environment.

Specifically, the task attraction of a chatbot lies in the ability of a chatbot to help users accomplish the task. Not all chatbots have high task attraction, especially when compared to humans. Spence et al. [60] examined differences when individuals view a broadcast weather forecast delivered by a professional, an amateur, or a social robot and found that individuals believe professional meteorologists are significantly more credible than amateur or robotic counterparts. Edwards et al. [12] investigated whether the message design logic of a robot affects a person’s evaluation of it.

The social attraction of a chatbot is a measure of its social ability, i.e., whether the chatbot can attract users to establish a social relationship with it or not. Discussions of the social attraction of chatbots are often addressed in related research. Spence et al. [61] found that people tend to have lower expectations of social attraction when interacting with bots compared to interacting with other people. Meanwhile, Sundar et al. [63] explored the social attraction of robots with different social behaviors and further analyzed the effect of robots’ social attraction on their usage intention. Hong and Xu [20] argued that it is by presenting themselves as if they were real humans that chatbots can establish substantive relationships with human users, and shape social interaction behaviors and relational patterns among online user groups based on the diffusion and dissemination of information.

Social attraction is a general assessment of the social ability of chatbots, while social attributes provide a more detailed examination of the social ability of chatbots. These constructs complement each other: social attraction captures users’ emotional motivation to engage, while social attributes reflect more specific evaluative criteria that shape relational trust. Incorporating both into the model allows for a more comprehensive understanding of how different facets of perceived sociality influence users’ para-social interaction and emotional support. Cuddy et al. [7] have proposed the Stereotype Content Model (SCM), which contains two dimensions: perceived warmth and perceived competence. The SCM model has been used to study stereotypes in human–robot interaction. Seiler and Schär [57] conducted an online survey and found that people perceived the chatbots according to the SCM so that companies can enhance a client’s chatbot experience by using insights from SCM. Carpinella et al. [4] created and validated the Robot Social Attributes Scale (RoSAS) based on the five dimensions of the Godspeed scale. The scale contains three dimensions: warmth, competence, and discomfort, each of which consists of six sub-items. This study will use perceived warmth and perceived competence to explore the social attributes of chatbots. Building on these theoretical insights, it is important to consider the empirical foundations of chatbot–human emotional interaction. Table 1 provides a summary of representative studies that examine user perceptions, emotional support, and behavioral outcomes in chatbot communication.

**Table 1:** Prior studies related to chatbot-human interactions

Study Focus	Source	Key Findings
Systematic review of human-chatbot interactions	Rapp, Curti, & Boldi [51]	Synthesizes 83 studies on chatbot-user interaction, highlighting trust, satisfaction, and emotional engagement
Parasocial relationships with chatbots	Skjuve, Følstad, Fostervold, & Brandtzaeg [58]	Explores one-sided emotional bonds users form with chatbots, and their effect on satisfaction
Media dependency and emotional interaction	Yuan, Cheng, & Duan [73]	Emotional interaction with chatbots increases users’ reliance on them as a form of social media
Emotional disclosure in mental health chatbot counseling	Park, Chung, & Lee [45]	Chatbot emotional disclosure enhanced user satisfaction and reuse intention, mediated by users’ disclosure intention and perceived intimacy
Parasocial relationship as mediator in chatbot continuance usage	Ramya & Alur [49]	Parasocial relationship mediates the effect of service quality on continued usage. Information and system quality foster Parasocial relationship among millennials and Gen Z users
Perceived authenticity in chatbot interactions	Khan, Tarofder, Gopinathan, & Haque [29]	Empathy, humanness, and warmth enhance perceived authenticity. Authenticity increases trust and satisfaction. Humor plays a complementary role
Consumer trust and emotional response to chatbots in marketing	Wang, Li, Fu, & Jin [68]	Chatbot interactions triggered more subconscious attention and emotional regulation but lower trust compared to human agents, especially under subjective tasks

Perceived competence is the presence or absence of competence traits related to the realization of a person's intentions, and can be characterized by intelligence, skill, creativity, efficacy, and independence [14]. Piçarra and Giger [48] found that the perceived competence of robots has a significant positive effect on the attitude toward working with the robot, positive or negative subjective norms, and the perceived behavioral control, which can be used to influence behavioral desire and behavioral intention towards working with the robots. Other scholars have argued that the perceived competence of chatbots can have a negative impact. Liu et al. [36] found that the perceived competence of robots increased rather than decreased concerns of older adults. Older adults have three main concerns about chatbots: technical issues, financial issues, and privacy issues.

Fiske et al. [14] argued that perceived warmth referred to whether a person was perceived to have positive intentions and tended to look after the interests of others. Within the warmth dimension, traits such as friendliness, helpfulness, sincerity, trustworthiness, and morality all contribute to perceived positive intentions. Perceived warmth and competence are not only conceptually distinct but also elicit different psychological and behavioral responses. For instance, consumers tend to perceive greater warmth in non-profit organizations and greater competence in for-profit organizations,furthermore, their purchasing behaviors are often driven by recognition of the competence of for-profit entities. This suggests that warmth is more closely associated with emotional or relational judgments, while competence is linked to performance-based evaluations and decision-making [1]. Perceived warmth and perceived competence are often used together in studies of chatbots. For example, Reeves et al. [53] found that warmth and competence are important in assessing chatbots, so the influence of these two factors in robot design should be considered. Mieczkowski et al. [42] also investigated the relationship between warmth and competence, the perception of emotional responses and behavioral tendencies in the context of chatbots.

Para-social interaction refers to the audience's reaction to the characters in the mass media as if they were real people Horton & Wohl.[21] The existence of para-social interaction was verified by studies on mass media in the 1970 s, especially after the formulation of the “uses and gratifications” theory, and research on para-social interaction has been gradually carried out [13]. In the age of intelligent media, parasocial interaction expanded the concept of regulatory fit and served as a trigger to activate anthropomorphic trust in social robots [59]. Pentina et al. [47] integrated parasocial Interaction with interpersonal relationship theories to propose an explanatory model that advances our understanding of the mechanism behind the development of human-AI relationships.

Attraction is crucial for relationships to progress [6]. Rubin et al. (1987) argued that social attraction, physical attraction and task attraction positively affect prosocial interactions. Human-like chatbots lead to greater satisfaction and trust among customers [25]. Zheng et al. [75] found that technology attraction positively affected the users’ parasocial interaction on social shopping websites. Houlberg [22] included attributes such as warm and friendly, honest, intelligent and educated, qualified and competent in his consideration of para-social interactions between audiences and TV anchors.

Based on the above literature review, the first hypothesis is proposed.

- H1a: Social and task attraction are positively associated with users’ para-social interaction with chatbots.
- H1b: Perceived warmth and competence are positively associated with users’ para-social interaction with chatbots.

Emotional support expresses understanding, encouragement, empathy, affirmation, recognition, sympathy and concern for others Liu et al. [37] Emotional support is a form of social support [74]. In the context of this study, emotional support refers to the support users perceive to receive from chatbots during interactions. When individuals share stressful events, the chatbot not only provides useful information but also sends messages expressing empathy, as humans normally do [15].

According to Duran and Kelly [11], communicative competence is most likely accomplished through a person’ s ability to empathize with others and demonstrate a sense of belonging and emotional support. And the level of communicative competence tends to influence others’ perceptions of social attraction, task attraction, and physical attraction of “me”. Huang and Gursoy [24] found that customers’ perceived emotional support from chatbots significantly influences their satisfaction with customer service. When chatbots are expected to provide emotional support, implicit mind perception becomes especially salient, as such support requires the perception that the agent is capable of understanding, experiencing, and responding to emotions [34]. Therefore, the second hypothesis is proposed.

- H2a: Social and task attraction are positively associated with users’ perceived emotional support from chatbots.
- H2b: Perceived warmth and competence are positively associated with users’ perceived emotional support from chatbots.

### Attractions of Chatbots, Usage Intention and Media Dependency on Chatbots

To predict users’ willingness to use chatbots, Robinson et al. (2018)examined the Robot Usage Intention scale (RUI). In this study, chatbots usage intention is defined as users’ subjective willingness and behavioral tendency to continue interacting with the chatbot. This construct reflects both attitudinal and motivational dimensions and has been widely used as a key outcome in human–AI interaction research. Usage intention reflects the long-term effectiveness and user acceptance of such systems. For elderly people’s usage intention of escort robots, perceived technical support as an external push has a direct impact on usage intention He & He [19]. At the same time, obtaining a sense of enjoyment, and enhancing pleasure or happiness through the companionship and interaction provided by the companion robot satisfies the emotional needs of the elderly for the companion robot, which in turn positively affects the willingness to use. Therefore, the third hypothesis is proposed.

- H3a: Social and task attraction are positively associated with users’ intention to use chatbots.
- H3b: Perceived warmth and competence are positively associated with users’ intention to use chatbots.

Media Dependency Theory (MDT), proposed by Ball-Rokeach and DeFleur, views media as part of a larger communication system composed of the audience, media, and society. According to MDT, individuals depend on media to achieve personal goals such as understanding, orientation, and social interaction. This dependency is dynamic and bidirectional, varying across media types and user contexts Jung.[28] Although originally developed to explain reliance on traditional mass media, MDT has evolved with the media landscape. In today’s digital environment, researchers have extended the theory to encompass diverse forms of media, including social media, mobile applications, and AI-based interactive systems. For example, Nawi et al. [43] applied MDT to examine youth dependency on new media platforms in Malaysia. In this study, media dependency refers to users’ reliance on chatbots as a new form of media. Chatbots, as interactive and responsive media agents, can fulfill both functional and affective roles, thereby forming dependency relationships consistent with the MDT framework. Prior studies have shown that media systems capable of emotional interaction may elicit greater user reliance and habitual use [10]. Therefore, the fourth hypothesis is proposed.

- H4a: Social and task attraction are positively associated with users’ media dependency on chatbots.
- H4b: Perceived warmth and competence are positively associated with users’ media dependency on chatbots.

### Para-social Interactions, Emotional Support, Usage Intention and Media Dependency on Chatbots

The effect of relationship type on customers’ para-social interactions found that customers’ relationships with services, brands, and other customers influenced their para-social interactions, which in turn positively influenced their usage intention of brands [33]. Meanwhile, para-social interactions are often used as mediating variables in related studies. Tsai et al. [65] found that the effect of chatbots’ higher social presence communication on consumer engagement outcomes was mediated by perceived para-social interactions and conversations in their study. Similarly, Xu et al. [71] demonstrated that tourists'acceptance of ChatGPT for travel-related services was significantly influenced by parasocial interactions, which mediated the relationship between social influence, perceived value, and user acceptance. Therefore, the fifth hypothesis is proposed.

- H5a: Para-social interaction mediates the relationship between chatbot attraction and users’ usage intention.
- H5b: Para-social interaction mediates the relationship between chatbot social attributes and users’ usage intention.

The degree of para-social interaction between users and chatbots has a significant positive effect on the media dependency of users [18]. Para-social interaction is often used as a mediating variable in related studies. In his study of the relationship between consumers and brands in social media environments, Labrecque [32] used para-social interaction as a mediating variable between openness, interactivity and willingness to share information, loyalty. Therefore, the sixth hypothesis is proposed.

- H6a: Para-social interaction mediates the relationship between chatbot attraction and users’ media dependency.
- H6b: Para-social interaction mediates the relationship between chatbot social attributes and users’ media dependency.

In their study of online health community users’ usage intention of information services, Wu and Li [70] found that both information support and emotional support had a significant positive effect on users’ usage intention. Emotional support is often used as a mediator. Huang and Gursoy [24] found that during the informational stage of customer decision-making, emotional support from chatbots using an abstract language style significantly mediated the effect of chatbot interaction on service satisfaction, emphasizing the context-specific value of emotional support in online service encounters. In addition, Meng and Dai [41] found that emotional support from AI chatbots significantly reduced users’ stress and anxiety, especially when the chatbot engaged in self-disclosure. Therefore, the seventh hypothesis is proposed.

- H7a: Perceived emotional support mediates the relationship between chatbot attraction and users’ usage intention.
- H7b: Perceived emotional support mediates the relationship between chatbot social attributes and users’ usage intention.

Vincent [66] argues that the cell phone is an emotional chatbot. Every interaction between a person and a cell phone involves e-emotion, which in turn makes people dependent on and attached to the electronic device. Emotional support is often used as a mediating variable. Jin and Wang [27], in their study of the effect of gratitude on adolescents’ engagement in learning, used teachers’ emotional support and students’ basic psychological needs as mediating variables. Therefore, the last hypothesis is proposed.

- H8a: Perceived emotional support mediates the relationship between chatbot attraction and users’ media dependency.
- H8b: Perceived emotional support mediates the relationship between chatbot social attributes and users’ media dependency.

**Fig. 1:**
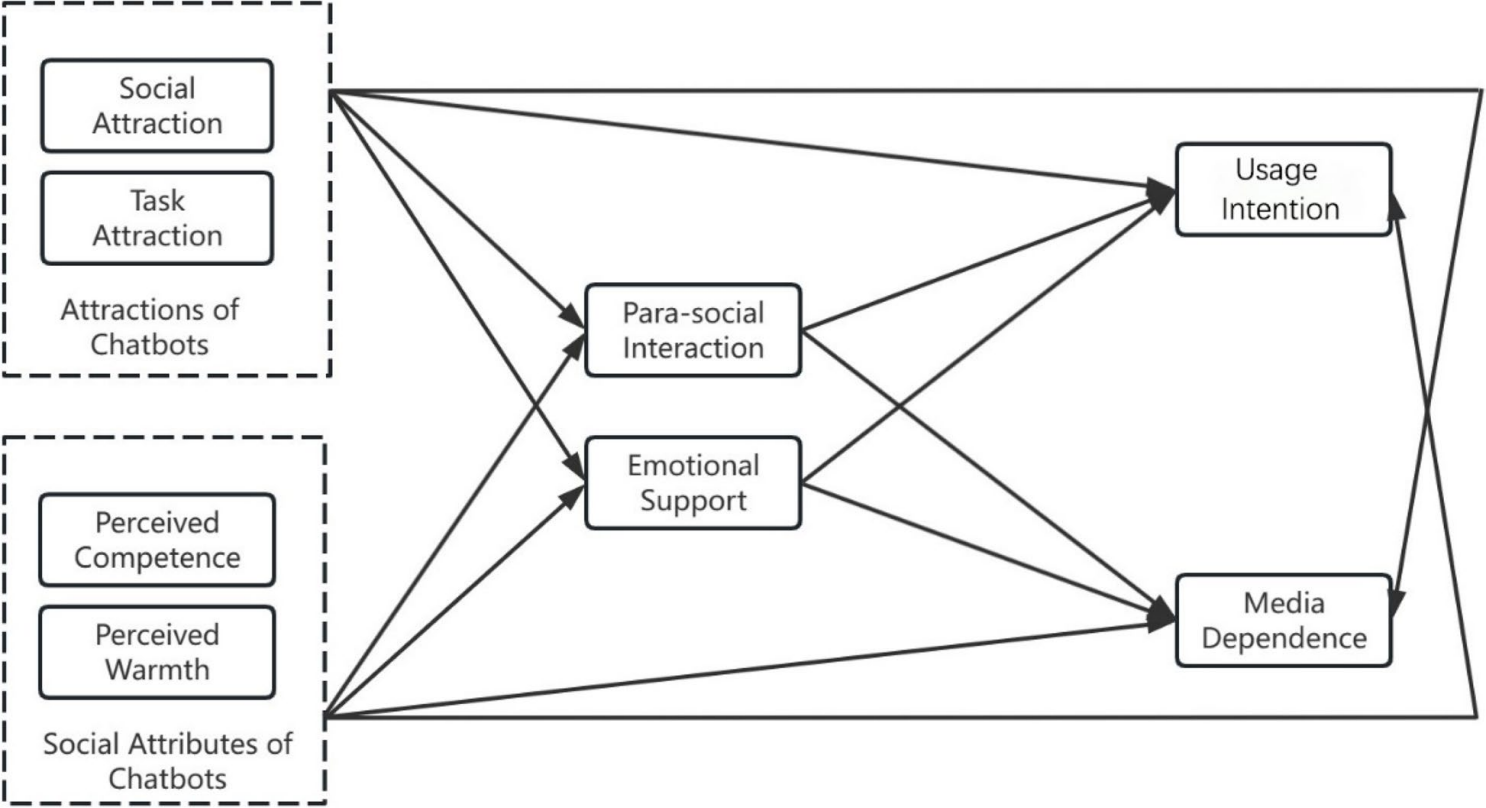
Conceptual model

The conceptual model of this study is shown in Fig. 1.

## Methods

### Research Object and Variable Measurement

ChatGPT is a public tool developed by OpenAI that is based on the GPT language model technology. As one of the most popular chatbots, many scholars only view it as a tool not an object of emotional communication. Although direct access in mainland China requires the use of a virtual private network (VPN), ChatGPT’s technological advancement and wide recognition among digitally literate populations support its selection as a representative research object for exploring human–AI interaction. This study presents the emotional connection through para-social interaction and emotional support in psychology and explores the effect of emotional connection on users’ media dependency and usage intention.

The questionnaire of this study contains two main parts. The first part is about demographic characteristics and whether or not ChatGPT has been used by participants. The second part is about the design of a scale to measure eight variables: Social attraction refers to the extent to which users perceive ChatGPT as socially likable and friendly, and is measured based on McCroskey and McCain’s [38] interpersonal attraction scale. Task attraction, also drawn from McCroskey et al.[40], captures users’ willingness to collaborate with ChatGPT to accomplish specific tasks. Perceived competence is operationalized as users’ assessment of ChatGPT’s reliability, informativeness, and ability to fulfill commands, based on items adapted from Carpinella et al. [4] and Fiske et al. [14]. Perceived warmth evaluates users’ impressions of ChatGPT’s kindness, sincerity, and emotional accessibility, measured following Fiske et al. [14] and Carpinella et al. [4]. Para-social interaction refers to the illusion of a reciprocal, affective bond formed with ChatGPT during use, and is measured using items adapted from Schramm and Hartmann [57]. Emotional support reflects the perceived empathy, encouragement, and emotional reassurance that users receive from ChatGPT, based on scales validated by Oh et al. [45] and Yoo et al. [73]. Usage intention assesses users’ willingness to continue engaging with ChatGPT, and to prefer it over real-world communication in certain scenarios, following Robinson et al. [55]. Media dependency is defined as the extent to which users rely on ChatGPT for functional and emotional needs, with reference to scales developed by Chan-Olmsted and Xiao [5]. All the variables were measured based on established scales and the detailed questionnaire items were shown in Table 2.

**Table 2:** Participant Demographics

Variables	Category	Frequency	Percentage
Gender	Male	769	49.5%
	Female	784	50.5%
Age	Under 18	149	9.6%
	18–29	528	34.0%
	30–41	303	19.5%
	42–53	343	22.1%
	54–65	180	11.6%
	66 and above	51	3.3%
Education	High school degree or below	272	17.5%
	Some college or associate degree	368	23.7%
	Bachelor's degree	666	42.9%
	Beyond bachelor’s degree	247	15.9%
Occupation	Full-time student	270	17.4%
	Production personnel	194	12.5%
	Sales personnel	208	13.4%
	Marketing/PR personnel	79	5.1%
	Customer service personnel	95	6.1%
	Administrative/logistics personnel	104	6.7%
	Human resources	174	11.2%
	Finance/audit personnel	160	10.3%
	Technical/R&D personnel	135	8.7%
	Management personnel	135	8.7%

### Data Collection

The questionnaire was distributed through the largest Chinese online survey platform SoJump.com (wjx.cn). Participant recruitment relied on non-probability sampling methods, combining convenience sampling and snowball sampling strategies. There was a disclaimer in the debriefing reminding the respondents that the study was a piece of academic research. Each respondent received 5 RMB through the SoJump system as an incentive for participation.

Informed consent to participate was obtained from all of the participants in the study Before the questionnaire was distributed, we obtained the consent of all the participants. At the beginning of the questionnaire, we stressed the anonymity and privacy protection and encouraged participants to exit any time they felt uncomfortable.

Before the formal distribution of questionnaires, 50 responses were collected for pre-survey, and the questionnaire was adjusted and improved based on the feedback from the survey. The formal survey was conducted in August 2023, and a total of 1589 responses were collected. The questionnaire link and WeChat QR code were distributed through convenience sampling and snowballing methods. The first question of the questionnaire, “Have you ever used ChatGPT”, was used to screen ChatGPT users.

According to Meade and Craig’s [40] recommendations, questionnaires with the same option (e.g., choice 1) throughout the whole questionnaire were excluded. 1553 valid responses were finally obtained, with a validity rate of 97.73%. Among them, 49.5% were male samples and 50.5% were female samples. In terms of age structure, 18–29 years old has the highest proportion, accounting for 33.4%, followed by 42–53 years old, accounting for 22.1%. Although full-time students were included in the sample, they only constituted 17.4% of all participants. The remaining respondents were working professionals from diverse occupations, such as production, sales, marketing, customer service, logistics, HR, finance, and R&D. This indicates that the sampling was not limited to student participants, but broadly representative of various social roles. Participant demographics are summarized in Table 3.

**Table 3:** Questionnaire (variable measurement)

Variables	Questionnaire Items	Sources
Social Attraction	Cronbach’ s alpha coefficient = 0.850; CR = 0.796; AVE = 0.369	McCroskey and McCain [38]
	I think ChatGPT could be my friend	
	I want to have a friendly conversation with ChatGPT	
	ChatGPT is delightful	
Task Attraction	Cronbach’ s alpha coefficient = 0.861; CR = 0.807; AVE = 0.513	McCroskey et al. [39]
	If I want to accomplish a task (e.g., provide information, a fun test, etc.), I can rely on ChatGPT	
	I enjoy working with ChatGPT	
	I would recommend ChatGPT to others as a workmate	
	ChatGPT takes its job very seriously	
Perceived Competence	Cronbach’ s alpha coefficient = 0.879; CR = 0.820; AVE = 0.479	Carpinella et al. [4], Fiske et al. [14]
	I think ChatGPT is reliable (e.g., can fulfill user commands, can provide accurate information, etc.)	
	I think ChatGPT is knowledgeable (e.g., can answer basically all questions, has a more powerful search function, etc.)	
	I think ChatGPT is responsive (e.g., quick to understand commands and respond, etc.)	
	I think ChatGPT as interactive (e.g., being able to have conversations, games, etc. with the user)	
Perceived Warmth	Cronbach’ s alpha coefficient = 0.831; CR = 0.758; AVE = 0.444	Carpinella et al. [4], Fiske et al. [14]
	I think ChatGPT is forgiving (e.g., rarely gets angry, calculating, etc.)	
	I think ChatGPT to be welcoming (e.g., responding positively to the user, rarely showing indifference, impatience, etc.)	
	I think ChatGPT is kind (e.g., helpful, understanding, etc.)	
	I think ChatGPT is genuine (e.g., answers questions honestly, treats people	
Para-social Interaction	Cronbach’ s alpha coefficient = 0.911; CR = 0.884; AVE = 0.418	Schramm & Hartmann [56]
	I think ChatGPT is like a normal and real person	
	ChatGPT seems to know what’ s on my mind	
	I can trust ChatGPT to give me information	
	I was interested in the information ChatGPT provided me with	
	If I see a story about ChatGPT in a newspaper, magazine, website, etc., I will go read the story	
	I would like to meet the real person behind ChatGPT in reality	
	If ChatGPT recommends a product to me while chatting with me, I’ ll go for it	
	If I don’ t talk to ChatGPT for a long time, I’ ll miss it	
	I get upset if ChatGPT doesn’ t understand my question correctly	
	When I end a chat with ChatGPT, I feel lost	
	I would find it offensive if someone said something bad about ChatGPT	
Emotional Support	Cronbach’ s alpha coefficient = 0.862; CR = 0.789; AVE = 0.431	Oh et al. [44], Yoo et al. [72]
	Talking to ChatGPT can be an encouragement to me	
	ChatGPT showed empathy for me during the chat	
	Talking to ChatGPT makes me feel relaxed	
	Talking to ChatGPT makes me feel not alone	
	ChatGPT is a great source of emotional support for me	
Usage Intention	Cronbach’ s alpha coefficient = 0.857; CR = 0.863; AVE = 0.677	Robinson et al. [54]
	I think about talking to ChatGPT when I’ m in a bad mood	
	I’ d feel like I was missing something if I didn’ t talk to ChatGPT for a few days	
	I’ d much rather chat with ChatGPT than have a real-world social interaction	
Media dependency	Cronbach’ s alpha coefficient = 0.914; CR = 0.821; AVE = 0.487	Chan-Olmsted &Xiao [5]
	I usually interact with ChatGPT	
	I would seek help from ChatGPT	
	I’ ll spend time on ChatGPT	
	I’ ll often ask ChatGPT to help me with tasks	
	I’ ll be interacting with ChatGPT for a long time	

^*^CR: Combined reliability; AVE: Average Variance Extracted

## Analysis and Results

### Reliability Test

The study first examined the internal consistency of the questionnaire by conducting reliability tests on eight variables: social attraction, task attraction, perceived competence, perceived warmth, para-social interaction, emotional support, usage intention, and media dependency. The Cronbach’α values of the total valid samples ranged from 0.831 to 0.914 for the eight variables. According to Nunnally’s standard Cronbach’s α value should be greater than 0.7 [23], so it can be confirmed that this sample has high reliability.

In this study, exploratory factor analysis was conducted using SPSS 26.0 and the results obtained are shown in Table 1. The results showed that the Kaiser–Meyer–Olkin (KMO) test statistics ranged between 0.731–0.829 and Bartlett's spherical test p-values were less than 0.001, indicating that the variables of the scale were significantly correlated. Using principal component analysis, 10 main factors with eigenroots greater than 1 were extracted from the scale with a cumulative variance contribution of 79.973%. Using maximum variance rotation, each question item had a loading value greater than 0.40 on the common factors, indicating that the overall reliability of the scale was high. The composite reliability (CR) values for all latent variables ranged between 0.758–0.884, which meets the acceptable threshold of CR value of 0.7 suggested by Raykov [52]. The average variance extracted (AVE) values ranged from 0.369–0.677, which is in line with the acceptable threshold of AVE value 0.36 suggested by Fornell and Larcker [16], indicating that the structural validity of the scale is acceptable. To assess discriminant validity, we applied the Fornell-Larcker criterion Fornell & Larcker [16], comparing the square root of the average variance extracted (AVE) for each construct with its correlations to other constructs. Results confirmed discriminant validity, as the square root of AVE for each construct (ranging from 0.61 to 0.82) exceeded all inter-construct correlations (ranging from 0.25 to 0.45). For instance, social attraction (√AVE = 0.82) showed higher within-construct validity than its correlations with other constructs (e.g., r = 0.41 with task attraction, r = 0.39 with perceived competence). Similar patterns held across all variables, ensuring that each latent construct was empirically distinct. Additionally, we computed heterotrait-monotrait (HTMT) ratios, all of which fell below the conservative threshold of 0.85 (range: 0.28–0.72), further supporting discriminant validity (Henseler et al., 2015). These results collectively confirm that the measurement model satisfies discriminant validity requirements.

### Hypothesized Paths Test

AMOS 26 was used to conduct a two-step structural equation model testing of the overall path of the proposed model. In the first step of the measurement phase, the work analyzed all the measurement items and examined the correlated residuals and cross-loadings for each item to confirm that they could be combined into indices following the original measurement scales. In the second step, the confirmatory structural equation model was used to test the relationships among variables. The resulting model had good fit: CMIN/DF = 2.284; RMSEA = 0.068; CFI = 0.966; IFI = 0.966; GFI = 0.954, NFI = 0941, in accordance with Bagozzi and Yi [3].

**Fig. 2:**
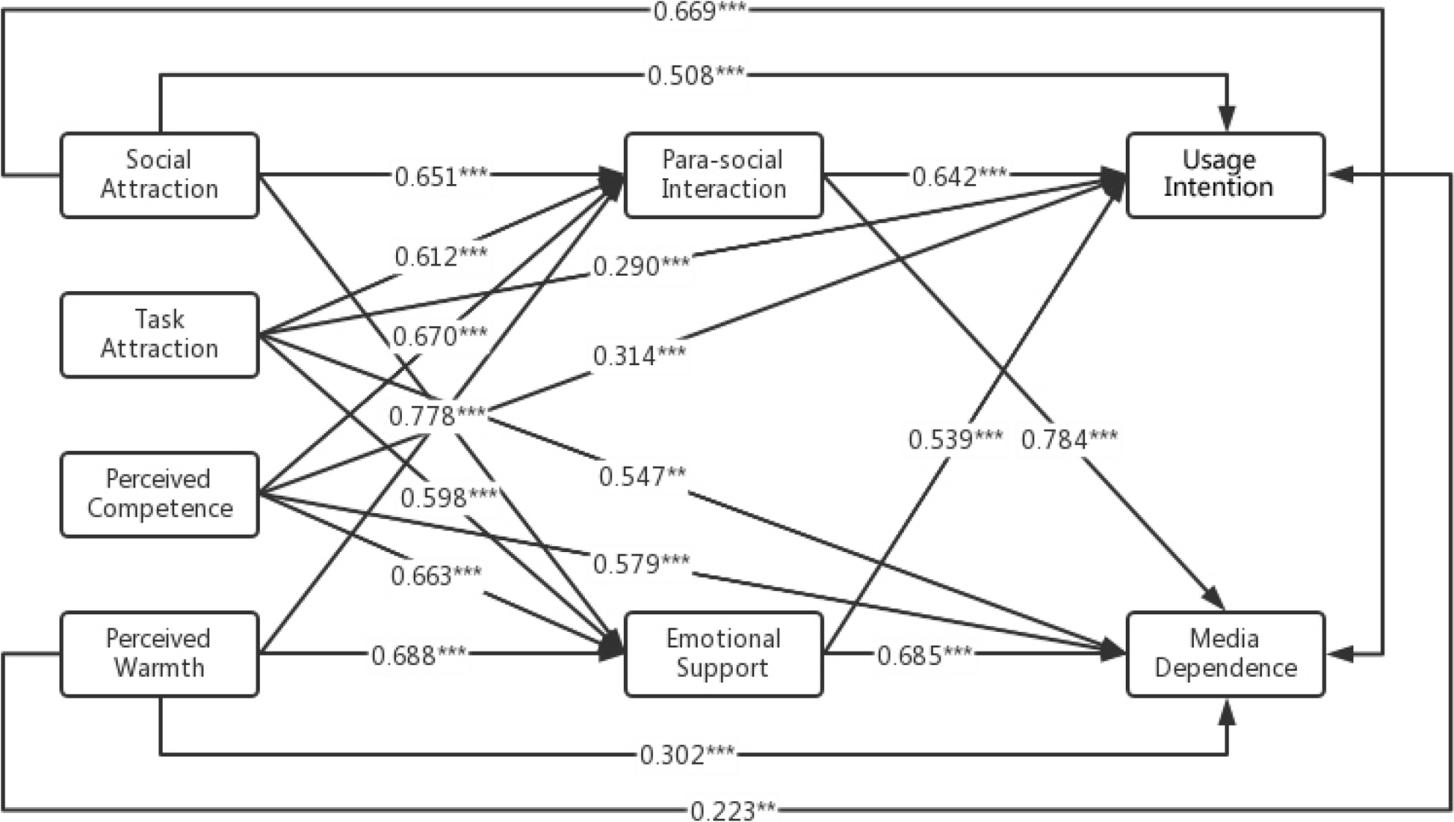
Statistical model. Note: All coefficients are standardized estimates. *p < 0.05, **p < 0.01, ***p < 0.001

The results in Fig. 2 show that social attraction and task attraction in chatbots’ attraction and perceived competence and perceived warmth in chatbots’ social attributes have a positive effect on the degree of para-social interaction between users and chatbots, H1a and H1b are supported, i.e., indicating that chatbots perceived as more socially engaging and functionally capable are more likely to elicit stronger para-social interaction from users.

Social attraction and task attraction in chatbots’ attraction as well as perceived competence and perceived warmth in chatbots’ social attributes have a positive effect on the emotional support of chatbots, thus H2a and H2b are supported, i.e., the more reliable and warmer chatbots are, the more chatbots can appease users’ emotions.

Social attraction and task attraction in chatbots’ attraction and perceived competence and perceived warmth in chatbots’ social attributes have a positive effect on users’ usage intention, thus H3a and H3b are supported, i.e., the more knowledgeable and friendlier chatbots are, the stronger users’ usage intention is.

Social attraction and task attraction in chatbot attraction, and perceived competence and perceived warmth in chatbots’ social attributes have a positive effect on users’ media dependency, thus H4a and H4b are supported, i.e., the more chatbots interact with users, the more intelligent and kinder they are, and the more users rely on the chatbots.

In addition, the degree of para-social interaction between users and the chatbots has a positive effect on the users’ usage intention, i.e., the higher the degree of para-social interaction between users and chatbots, the stronger users’ usage intention of chatbots. The degree of para-social interaction between users and chatbots has a positive effect on the user’s media dependency, i.e., the higher the degree of para-social interaction between users and chatbots, the more the users rely on the chatbots. The emotional support of chatbots has a positive effect on the users’ usage intention, i.e., the more chatbots can encourage and support users, the stronger users’ usage intention of chatbots. Emotional support of chatbots has a positive effect on users’ media dependency, i.e., the more chatbots can calm users’ emotions, the more users rely on chatbots.

### Tests for Mediating Effects

Mediation analysis was conducted using AMOS 26. The number of bootstrap resamples was set to 5000, and 95% bias-corrected confidence intervals were calculated for all indirect effects. In the structural equation model, direct paths from the three stimulus variables to the three response variables were retained to control for direct effects during mediation testing. If the confidence intervals did not contain 0, statistical significance was indicated. Table 4 demonstrates the details of the confidence intervals for the mediating effects, which are significant for all mediating paths, i.e., hypotheses H5a to H8b are all supported.

**Table 4:** Results of the mediation effects test

Mediated Effect	Estimation	SE	95% confidence interval	
			Lower2.5%	Capped2.5%
Social Attraction → Para-social Interaction → Usage Intention	0.6592^***^	0.022	0.6157	0.7028
Task Attraction → Para-social Interaction → Usage Intention	0.5687^***^	0.031	0.5073	0.6301
Perceived Competence → Para-social Interaction → Usage Intention	0.6151^***^	0.029	0.5581	0.6722
Perceived Warmth → Para-social Interaction → Usage Intention	0.4164^***^	0.032	0.3526	0.4801
Social Attraction → Para-social Interaction → Media dependency	0.6592^***^	0.022	0.6157	0.7028
Task Attraction → Para-social Interaction → Media dependency	0.5687^***^	0.031	0.5073	0.6301
Perceived Competence → Para-social Interaction → Media dependency	0.6151^***^	0.029	0.5581	0.6722
Perceived Warmth → Para-social Interaction → Media dependency	0.4164^***^	0.032	0.3526	0.4801
Social Attraction → Emotional Support → Usage Intention	0.7117^***^	0.025	0.6636	0.7597
Task Attraction → Emotional Support → Usage Intention	0.6042^***^	0.035	0.5364	0.6720
Perceived Competence → Emotional Support → Usage Intention	0.6631^***^	0.032	0.6005	0.7258
Perceived Warmth → Emotional Support → Usage Intention	0.6499^***^	0.029	0.5924	0.7073
Social Attraction → Emotional Support → Media dependency	0.7117^***^	0.025	0.6636	0.7597
Task Attraction → Emotional Support → Media dependency	0.6042^***^	0.035	0.5364	0.6720
Perceived Competence → Emotional Support → Media dependency	0.6631^***^	0.032	0.6005	0.7258
Perceived Warmth → Emotional Support → Media dependency	0.6499^***^	0.029	0.5924	0.7073

All coefficients are standardized estimates. *p < 0.05, **p < 0.01, ***p < 0.001

The results of all hypothesis tests are presented in Table 5. Based on the above analysis, a conceptual model is proposed to describe how chatbot attraction and social attributes are linked to users’ usage intention and media dependency. Chatbots’ machine learning, neural networks, intelligent algorithms and emotional arousal strategies have entered people's daily lives and are widely used in emotional companionship, entertainment and leisure. The qualities of chatbots including attraction and social attributes significantly affect users’ willingness to use and media dependency through the degree of users’ para-social interactions with chatbots and the emotional support of chatbots. The users’ degree of para-social interaction with chatbots and chatbots’ emotional support mediate the relationship between social attraction, task attraction, perceived competence, perceived warmth of the chatbot and the users’ usage intention and media dependency. The stronger the chatbots’ attraction and social attributes, the stronger the degree of para-social interaction between users and chatbots and the emotional support of the chatbots, resulting in a stronger usage intention of and media dependency on chatbots. It means that improving chatbots’ attraction and social attributes is conducive to enhancing the degree of para-social interaction between users and chatbots and the emotional support of the chatbot, thus increasing the users’ usage intention and media dependency.

**Table 5:** Hypothesis Test Results

Hypotheses	Results
H1a: Social and task attraction are positively associated with users’ para-social interaction with chatbots	Supported
H1b: Perceived warmth and competence are positively associated with users’ para-social interaction with chatbots	Supported
H2a: Social and task attraction are positively associated with users’ perceived emotional support from chatbots	Supported
H2b: Perceived warmth and competence are positively associated with users’ perceived emotional support from chatbots	Supported
H3a: Social and task attraction are positively associated with users’ intention to use chatbots	Supported
H3b: Perceived warmth and competence are positively associated with users’ intention to use chatbots	Supported
H4a: Social and task attraction are positively associated with users’ media dependency on chatbots	Supported
H4b: Perceived warmth and competence are positively associated with users’ media dependency on chatbots	Supported
H5a: Para-social interaction mediates the relationship between chatbot attraction and users’ usage intention	Supported
H5b: Para-social interaction mediates the relationship between chatbot social attributes and users’ usage intention	Supported
H6a: Para-social interaction mediates the relationship between chatbot attraction and users’ media dependency	Supported
H6b: Para-social interaction mediates the relationship between chatbot social attributes and users’ media dependency	Supported
H7a: Perceived emotional support mediates the relationship between chatbot attraction and users’ usage intention	Supported
H7b: Perceived emotional support mediates the relationship between chatbot social attributes and users’ usage intention	Supported
H8a: Perceived emotional support mediates the relationship between chatbot attraction and users’ media dependency	Supported
H8b: Perceived emotional support mediates the relationship between chatbot social attributes and users’ media dependency	Supported

## Discussion

### General Discussion

Based on social attractiveness theory, stereotype content model, para-social interaction theory and emotional support theory, this study builds a model of the impact of the emotional connection between chatbots and users on users’ media dependence and usage intention.

These findings are generally consistent with and reinforce prior research. H1a–H2b are supported by Rubin et al. [55], Zheng et al. [76], and Houlberg [23], who emphasize attraction and social cues as key drivers of para-social interaction and emotional connection. H3a–H4b align with Robinson et al. [55] and Nawi et al. [44], affirming that both social motivation and emotional affordances of chatbots enhance users’ intention and dependency.

Mediation effects proposed in H5a–H8b are consistent with Tsai et al. [65], Xu et al. [71], Labrecque [32], and Huang and Gursoy [24], who demonstrate the roles of para-social interaction and emotional support as key relational bridges in AI-user engagement. These findings collectively validate the integrated model developed in this study.

High perceived attraction and favorable perceived social attributes of chatbots can enhance users’ para-social interaction with them, thereby increasing the extent to which users feel emotionally supported. In users’ daily experience, chatbots such as ChatGPT are often used for translation, question answering, and other utility tasks. Therefore, users’ perception of the chatbot’s ability to understand commands and perform tasks plays a key role in their willingness to sustain para-social interaction.

When users perceive stronger emotional support from chatbots, they tend to show greater willingness to use such tools and exhibit higher levels of media dependence. Dou [10] believes that chatbots not only have a certain human-like intelligence but also can imitate human feelings. It is through the emotional support of the user that the chatbot represented by ChatGPT can establish a para-social interaction with people, thus increasing users’ willingness to use and media dependency.

A stronger degree of para-social interaction between users and chatbots can lead to a stronger willingness to use and media dependency on chatbots. A higher perceived degree of para-social interaction reflects users’ tendency to anthropomorphize chatbots, treating them more like real social actors in their subjective experience. As this study stated in the previous section, task attraction, social attraction, perceived competence, and perceived warmth are all positively correlated with the para-social interactions between chatbots and users, and ChatGPT’s success in precisely these points facilitates its para-social interactions with users.

With the anthropomorphic characteristics of physical social machines coming to the fore, people no longer need to imagine in their minds the characters set by robot vendors but rather set the real and touchable chatbots in front of them as living beings. The convenience of this digital interaction and the sense of control over intimate relationships will further enhance the role of chatbots in emotional support, and media dependency will become the behavioral basis for widespread penetration into human social practice, thus generating a powerful force for social change based on social synergy and commonality.

### Theoretical Implications

This study makes several contributions to the theoretical understanding of human–chatbot emotional interaction. First, by integrating Social Attractiveness Theory, the Stereotype Content Model, Para-social Interaction Theory, and Social Support Theory, the study offers a comprehensive framework to explain how users’ perceptions of attraction and social attributes shape their emotional connection with chatbots. Second, the findings extend existing para-social interaction literature-previously focused on media figures-by applying it to interactive AI agents, thus supporting the applicability of PSI theory in human-AI contexts. Third, emotional support, originally conceptualized in human-to-human interaction, is validated here as a meaningful mediating factor in human-chatbot relations, enriching the operationalization of social support in human–computer interaction studies.

Although the results of this study demonstrate that the emotional connection between chatbots and users has a positive impact on users’ media reliance and willingness to use, which has some implications for the future development of chatbots, it does not mean that we can ignore the “emotional pitfalls” involved. Many scholars have noted that the emotion between robots and users is unidirectional. Dou [10] argued that the emotions of chatbots towards humans belong to artificial emotions, which are a function of their artificial emotions. Unlike real emotions in human interaction, artificial emotions may occupy an advantageous and powerful position, which is not only deceptive but even kidnapping. Su [62] also mentioned that because humans can develop robots with emotions, people are concerned that these emotions may have long-term effects on an individual's emotions, especially when facing emotional risks[]. In the future development of chatbots, it is also important to be wary of venturing into the future by ignoring the emotional risk and carefully considering\ the ethical issues that are likely to arise.

### Practical Implications

This study has practical implications for the design and application of chatbots as well as for improving the interactions between users and chatbots.

With the continuous improvement of chatbots in anthropomorphic features and functions, chatbots can carry out social activities and emotional labor by following predefined procedures and rules in line with human psychological expectations, and users gradually forget the “performance” traits of chatbots as mediators and develop a deeper level of dependency on chatbots. Although the intelligence and autonomy of chatbots have been significantly improved with the support of emotional computing research, they are still weak artificial intelligence and technically immature. The willingness to use chatbots and the deepening of media dependency may allow users’ emotions to be manipulated by business and politics, which is worth pondering. It is crucial to adapt to the human–robot symbiosis environment in the era of smart media and improve their media literacy. Users should give full play to their subjective position, handle the human–robot relationship well, pay attention to the algorithmic and symbolic control behind the chatbots, and grasp the amount and degree of emotional compensation exchange to prevent over-personalization and avoid complete instrumentalization.

From a developer’s perspective, the findings suggest that chatbot interaction strategies should dynamically adapt to users’ emotional and task-oriented needs. For example, during informational tasks, emphasizing warmth and social attraction—such as through polite, empathetic, or engaging language—may help increase perceived emotional support and foster para-social interaction. In contrast, transactional stages may require greater signaling of competence and functionality to boost trust and satisfaction. Developers should also implement adaptive feedback loops that assess user engagement and modulate the tone or interaction style accordingly.

For platform operators and organizations, it is advisable to segment chatbot services based on user intent and emotional needs. Service types such as companionship, consultation, and task assistance may benefit from different affective strategies. Institutions should deploy scenario-specific interaction modules that integrate multimodal language design—textual, visual, auditory, and even implicit cues like body language—to more closely mirror natural human communication. Governance frameworks should also be established to monitor affective manipulation risks, ensuring ethical emotional design and transparency.

Meanwhile, as interpersonal relationships become increasingly fragmented, users tend to interact with chatbots with clear instrumental or emotional goals. In this regard, social chatbots can serve as a supplement to human interaction and help meet psychological and emotional needs. To improve their role in emotional companionship, chatbot systems should continuously optimize their natural language processing capabilities and emotional symbolic expression, and expand their semantic libraries by drawing on data from social media, online forums, and everyday discourse. Additionally, multimodal learning should be enhanced to support richer symbolic communication—such as facial expressions, emoji use, and situational understanding—which brings chatbots closer to human interaction patterns. Ultimately, chatbot design should remain human-centered, focusing on emotional authenticity, adaptability, and responsible affective design.

### Limitations and Suggestions for Future Research

This study has several limitations that should be acknowledged. First, the research employed a cross-sectional design, which restricts the ability to make causal inferences about the relationships among variables. Future research could adopt longitudinal or experimental designs to validate the directional effects and test causality. Second, the data were collected through self-reported questionnaires, which may introduce social desirability bias or response bias, especially in evaluating emotional constructs such as perceived emotional support and para-social interaction. Future studies could consider incorporating behavioral or physiological measures (e.g., eye-tracking, usage logs) to triangulate user responses. Third, this study focused exclusively on ChatGPT as the representative chatbot. Although it is a widely used and advanced system, relying on a single platform may limit generalizability. Moreover, the novelty of ChatGPT and its perceived technological sophistication may have elicited novelty bias, potentially inflating users’ emotional responses or usage intention. Future studies should replicate these findings across diverse chatbot platforms with varying degrees of anthropomorphism, task orientation, and user familiarity. Fourth, although this study was grounded in para-social interaction theory and media dependency theory, and measured key predictors such as social attraction, task attraction, perceived competence, and emotional support, other potentially important factors were not considered. These may include the degree of anthropomorphization, user empathy toward chatbots, or trust in algorithmic governance. Future research can explore additional explanatory mechanisms and extend the model with new variables or moderating factors. Lastly, expanding the sample size and ensuring greater demographic and contextual diversity (e.g., different cultures, user expertise levels, or chatbot application scenarios) will further improve the external validity of future studies.

## Data Availability

The data that support the findings of this study are available from the first author, Ke Zhang, upon reasonable request.
